# Evidence for Autoinduction and Quorum Sensing in White Band Disease-Causing Microbes on *Acropora cervicornis*

**DOI:** 10.1038/srep11134

**Published:** 2015-06-05

**Authors:** Rebecca H. Certner, Steven V. Vollmer

**Affiliations:** 1 Department of Marine and Environmental Sciences, Northeastern University, Nahant, Massachusetts, USA

## Abstract

Coral reefs have entered a state of global decline party due to an increasing incidence of coral disease. However, the diversity and complexity of coral-associated bacterial communities has made identifying the mechanisms underlying disease transmission and progression extremely difficult. This study explores the effects of coral cell-free culture fluid (CFCF) and autoinducer (a quorum sensing signaling molecule) on coral-associated bacterial growth and on coral tissue loss respectively. All experiments were conducted using the endangered Caribbean coral *Acropora cervicornis*. Coral-associated microbes were grown on selective media infused with CFCF derived from healthy and white band disease-infected *A. cervicornis*. Exposure to diseased CFCF increased proliferation of *Cytophaga-Flavobacterium* spp. while exposure to healthy CFCF inhibited growth of this group. Exposure to either CFCF did not significantly affect *Vibrio* spp. growth. In order to test whether disease symptoms can be induced in healthy corals, *A. cervicornis* was exposed to bacterial assemblages supplemented with exogenous, purified autoinducer. Incubation with autoinducer resulted in complete tissue loss in all corals tested in less than one week. These findings indicate that white band disease in *A. cervicornis* may be caused by opportunistic pathogenesis of resident microbes.

Worldwide coral epizootics have escalated in incidence and severity over the last 30 years and have contributed to the unprecedented decline in global coral reef health^1^,^2^. Despite the devastating effects of coral disease, key details regarding their etiology and ecology remain unknown. A prime example of one such disease is white band disease (WBD), an epidemic which has nearly eradicated two of the Caribbean’s most important reef-building corals^1^. Since 1979, WBD has decimated 95% of regional *Acropora* populations, resulting in the listing of both *Acropora cervicornis* (Lamarck, 1816), staghorn coral and *Acropora palmata* (Lamarck, 1816), elkhorn coral as critically endangered on the IUCN Red List of Threatened Species^3^. Although its etiological agent has not been identified, the WBD phenotype is well documented across the Caribbean^4^,^5^. Like most coral diseases, it is characterized by rapid tissue loss as the disease progresses along a coral branch^1^.

Growing evidence indicates that WBD—and many other coral diseases—may be the result of opportunistic pathogenesis of resident coral bacteria^4^. Corals are host to dynamic and complex microbial communities that vary greatly within and between species^6^,^7^. These microbes, along with symbiotic dinoflagellates and a variety of other metazoans, constitute the coral holobiont^8^. Abiotic stressors, such as increased water temperatures, can heavily impact the coral holobiont, disrupting the balance of various host-symbiont relationships^9^. One of the hallmarks of coral disease is a shift in the composition of coral-associated microbial communities from symbionts to opportunistic species^4^,^9^. It has been postulated that during times of sustained environmental stress, certain bacteria take advantage of their vulnerable coral host and outcompete commensal microbes^4^. This phenomenon, known as the compromised-host hypothesis, suggests that the increasing incidence of coral disease is not due to the introduction of novel pathogens or increased pathogen virulence but rather results from the inability of susceptible corals to fight off infection^10^,^11^,^12^. Recent research has implicated vibrios as likely opportunistic pathogens, as diseased corals often host bacterial populations dominated by vibrios^13^,^14^. In addition, many *Vibrio* species engage in a phenomenon called quorum sensing (QS) as a regulator of virulence in a variety of hosts, potentially including corals^14^,^15^,^16^,^17^.

Quorum sensing refers to the mechanism by which populations of bacteria regulate gene expression through the concentration-dependent detection of signaling molecules called autoinducers^18^. It is a finely-tuned cell-cell communication system designed to coordinate the behaviour of a group of conspecifics and is stimulated by a growing bacterial population^18^. Bacteria synthesize autoinducers, which diffuse out of the cell and accumulate in the extracellular environment until their concentration crosses a detection threshold^19^. Bacterial recognition of these extracellular autoinducers initiates a signaling cascade resulting in the regulation of certain population-based actions controlled by gene expression. This allows bacterial populations to synchronize their behaviour and act collectively. QS has been shown to regulate a variety of important metabolic functions including biofilm formation, virulence, antibiotic production, motility, and sporulation^18^,^20^.

In general, autoinducers are classified as either species-specific (AI-1) or universal (AI-2), the former used by a narrow, closely-related group of bacteria while the latter is used by many gram-negative and gram-positive species^18^. One of the best-characterized species-specific AI-1 QS systems was first studied in gram-negative gammaproteobacteria and relies on acylated homoserine lactones (AHLs) as autoinducers^19^. AHLs belong to a class of neutral lipid molecules composed of an acyl chain of varying lengths and a homoserine lactone ring^21^. N-Hexanoyl-DL-homoserine lactone, the autoinducer used in this study, is a mid-sized molecule containing an acyl chain of six carbon atoms^22^. It is a common, virulence-associated AHL produced by a number of gram-negative bacteria including species of the genera *Pseudomonas*, *Aeromonas*, and *Serratia*^23^,^24^. N-Hexanoyl-DL-homoserine lactone is also employed by a variety of *Vibrio* species^25^,^26^. Numerous coral-associated bacteria, including many vibrios, have been shown to produce AHLs as part of their metabolism, although the connection between QS and coral disease has yet to be characterized^14^,^27^,^28^.

Here we explore the effects of (1) cell-free culture fluids (i.e. filtered coral homogenates) from diseased and healthy corals on coral-associated bacteria and (2) an established QS autoinducer on the transmission and progression of WBD-associated disease characteristics in a healthy *A. cervicornis* microbiomes. We extracted cell-free culture fluid (CFCF) from diseased and healthy coral homogenates in order to determine whether CFCF induces or inhibits the growth of *Cytophaga-Flavobacterium* and *Vibrio* spp., two groups associated with WBD and known to employ QS systems^16^,^25^,^29^,^30^,^31^. Next, we exposed *A. cervicornis* to N-Hexanoyl-DL-homoserine lactone (AHL) in controlled tank environments to determine if exogenous AHLs can induce WBD-like disease signs (i.e. tissue loss) in otherwise healthy corals.

## Results

### Selective Culturing of Coral-Associated Bacteria

**Figure 1:**
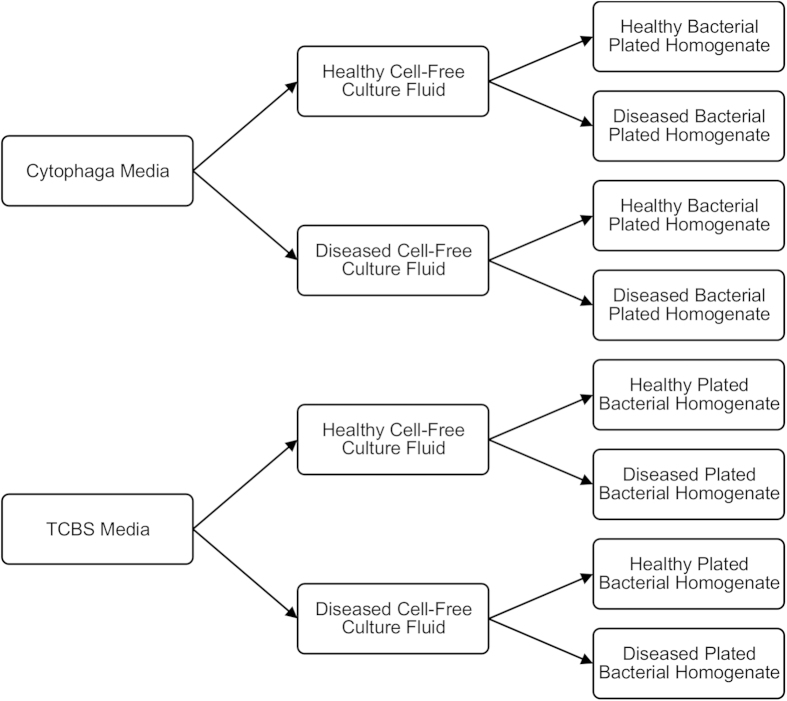
Experimental design for selective culturing of coral-associated bacteria. Diseased and healthy CFCF were added to both Cytophaga (*Cytophaga-Flavobacterium-*selective) and TCBS (*Vibrio*-selective) media resulting in four combinations of media/CFCF: Cytophaga medium/healthy CFCF, Cytophaga medium/diseased CFCF, TCBS medium/healthy CFCF, and TCBS medium/diseased CFCF. Diseased and healthy *A. cervicornis* bacterial homogenates were then diluted 1:10 with filtered seawater and plated onto all four media/CFCF combinations.

Coral-associated bacterial assemblages from WBD-infected and healthy *A. cervicornis* were plated onto selective media infused with cell-free culture fluid (Fig. 1). CFCF was obtained by filtering diseased and healthy coral tissue homogenates at 0.2 μm. Plated bacterial homogenates were obtained by filtering coral tissue homogenates at 5 μm to remove *A. cervicornis* cells and symbiotic dinoflagellates.

**Figure 2:**
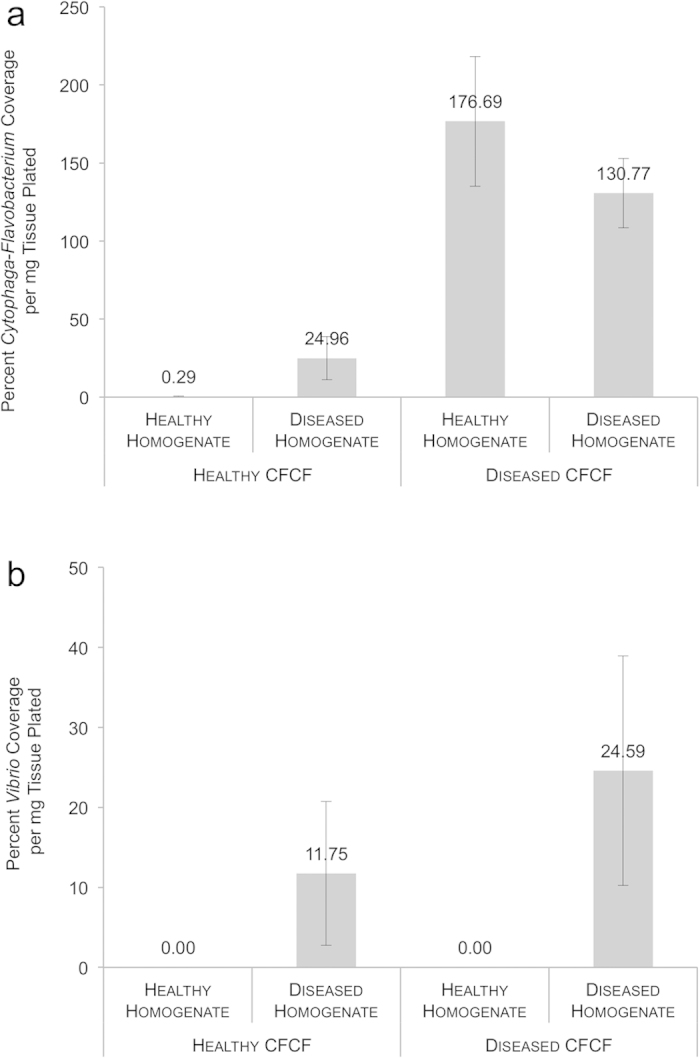
Bacterial growth for each CFCF/bacterial homogenate combination for *Cytophaga-Flavobacterium* (**a**) **and** ***Vibrio*** (**b**) **species**. Growth was measured by determining the percent bacterial coverage of each plate per mg of coral tissue plated using the point intercept method of counting in ImageJ^56^. Growth was analysed with a two-way ANOVA that considered disease state of the CFCF and disease state of the bacterial homogenate as fixed effects. Standard error bars are shown.

Origin of the CFCF (diseased versus healthy) had a significant effect on *Cytophaga*-*Flavobacterium* growth, which was defined as percent bacterial coverage per mg coral tissue plated (ANOVA, p = 0.0004) (Fig. 2a) (Supplementary Table S1). Disease state (diseased versus healthy) of the plated bacterial homogenate did not have a significant effect on *Cytophaga-Flavobacterium* growth (ANOVA, p = 0.6770) (Fig. 2a) (Supplementary Table S1). The effects of CFCF and plated bacterial homogenate were additive (p = 0.1890). Across healthy plated bacterial homogenates, the addition of CFCF from diseased corals to Cytophaga medium yielded significantly greater *Cytophaga*-*Flavobacterium* growth than did the addition of healthy CFCF (Tukey HSD, p = 0.0042) (Fig. 2a).

608% more *Cytophaga-Flavobacterium* growth was observed per mg healthy coral tissue when plated onto diseased CFCF as opposed to healthy CFCF (Fig. 2a). We found almost no culturable *Cytophaga-Flavobacterium* within healthy bacterial homogenates when grown in the presence of healthy CFCF (Fig. 2a). Although the effect was not significant, diseased bacterial homogenates grown in the presence of healthy CFCF yielded more *Cytophaga-Flavobacterium* growth—an 85% increase in percent coverage—than did healthy bacterial homogenates grown in the presence of healthy CFCF (Fig. 2a). Similarly, diseased bacterial homogenates grown in the presence of healthy CFCF yielded less *Cytophaga-Flavobacterium* growth—an 81% decrease—than did diseased bacterial homogenates grown in the presence of diseased CFCF (Tukey HSD, p = 0.0625).

CFCF origin (diseased versus healthy) did not have an effect (ANOVA, p = 0.8697) on *Vibrio* growth (Fig. 2b) (Supplementary Table S2). Only the disease state of the plated bacterial homogenate had a significant effect on percent bacterial coverage per mg coral tissue (ANOVA, p = 0.0232) (Fig. 2b) (Supplementary Table S2). Regardless of CFCF, diseased plated homogenates produced significantly more bacterial growth than did healthy plated homogenates on TCBS media. Healthy plated homogenates produced no culturable *Vibrio* spp. (Fig. 2b).

### AHL Addition Experiment

**Figure 3:**
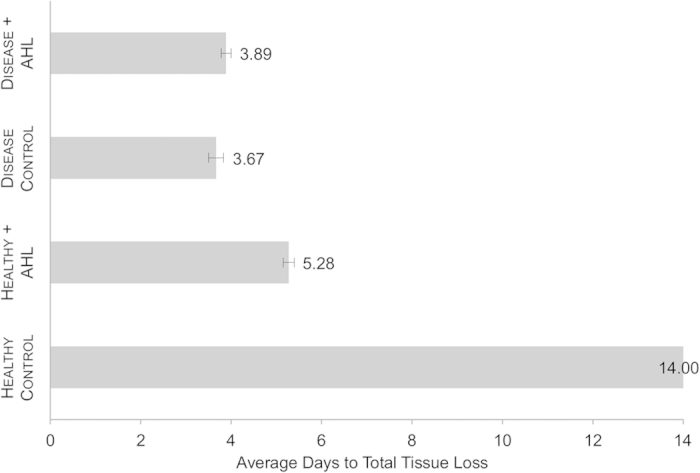
Time to mortality in healthy *A*. *cervicornis* exposed to coral tissue homogenates supplemented with AHL. Tissue homogenates of WBD-infected and healthy *A. cervicornis* were incubated with N-Hexanoyl-DL-homoserine lactone. Aquaria were then converted to closed systems and healthy test *A. cervicornis* were dosed with one of the four tissue homogenate/AHL combinations: Healthy + AHL, Disease + AHL, Healthy Control, and Disease Control. After dosage, the experiment was checked every 12 hours and the health status of each coral fragment was recorded. We equated total tissue loss with coral mortality. Time to coral mortality was analysed with a two-way ANOVA that considered disease state of the dosed tissue homogenate and addition or lack of AHL as fixed effects. Standard error bars are shown.

36 healthy *A. cervicornis* fragments were exposed to four combinations of WBD- and healthy-associated coral homogenates incubated with the either the AHL N-Hexanoyl-DL-homoserine lactone (Sigma Aldrich) or DI. N-Hexanoyl-DL-homoserine lactone was chosen based on the length of its acyl chain as six carbon AHLs are produced by multiple bacterial species, including a number of known marine *Vibrio* pathogens^14^,^22^. All coral fragments within the Healthy Control treatment remained healthy (no tissue loss) for the entire duration of the experiment (14 days) (Fig. 3). All corals in both the Disease Control and the Disease + AHL treatments experienced tissue loss consistent with WBD signs (tissue sloughing beginning around the lesioned area and traveling up the fragment) and total mortality within three to four days (Fig. 3). Similarly, all *A. cervicornis* fragments in the Healthy + AHL treatment experienced tissue loss and experienced total mortality within 4.5 to 5.5 days.

Disease state of the tissue homogenate had a significant effect on the average number of days to total tissue loss in healthy *A. cervicornis* (ANOVA, p < 0.0001) (Fig. 3) (Supplementary Table S3). Addition of AHL to tissue homogenates versus lack of AHL also produced a significant effect on days to tissue loss (ANOVA, p < 0.0001) (Fig. 3) (Supplementary Table S3). The two disease treatments (Disease Control versus Disease + AHL) yielded statistically equivalent effects on tissue loss (Tukey HSD, p = 0.5434) (Fig. 3). However, the two healthy treatments (Healthy Control versus Healthy + AHL) produced significant differences in time to tissue loss in the test corals (Tukey HSD, p < 0.0001) as fragments in the Healthy Control treatment displayed no disease symptoms whatsoever (Fig. 3). The AHL addition treatments (Healthy + AHL versus Disease + AHL) also yielded a significant effect on coral tissue loss (Tukey HSD, p < 0.0001) (Fig. 3). On average, the Healthy + AHL treatment took 26% longer to kill the healthy coral fragments compared to the Disease + AHL treatment.

## Discussion

*Cytophaga-Flavobacterium* spp. within bacterial homogenates derived from healthy *A. cervicornis* grew significantly more in the presence of cell-free culture fluid obtained from diseased corals than in the presence of CFCF obtained from healthy corals (Fig. 2a). This result indicates that diseased CFCF may enhance the proliferation of coral-associated *Cytophaga-Flavobacterium* either by containing molecules that induce growth and/or by lacking molecules that suppress growth. This finding is consistent with recent research suggesting that opportunistic pathogenesis of resident microbes can develop in vulnerable corals^4^,^9^. Diseased corals exist in a state of severe physiological stress, which may destabilize the relationship between the coral host and its microbial symbionts. As a result, commensal bacteria may turn pathogenic as they take advantage of their host’s decreased resistance to infection^11^,^12^,^32^. Our results support the compromised-host hypothesis in that *Cytophaga-Flavobacterium* spp. are present in small numbers on healthy corals but are able to grow unchecked when exposed to diseased coral CFCF^12^,^32^ (Fig. 2a). To our knowledge, this phenomenon has never before been demonstrated with coral-associated *Cytophaga-Flavobacterium* as it is usually linked with *Vibrio* species^4^,^9^.

Not only do our results demonstrate that diseased CFCF induces *Cytophaga-Flavobacterium* growth, they also imply that healthy CFCF may inhibit the growth of bacteria in this group. The growth of *Cytophaga-Flavobacterium* within diseased *A. cervicornis* bacterial homogenates appears to be hindered by CFCF obtained from healthy corals (Fig. 2a). Compared to diseased bacterial homogenates grown on diseased CFCF, diseased bacterial homogenates grown on healthy CFCF show a trend toward a significant (81%) decrease in *Cytophaga-Flavobacterium* growth (Fig. 2a). Inhibition of disease-associated microbes by healthy coral CFCF is consistent with recent findings that healthy corals are able to suppress the growth of potential pathogens via their symbiotic microbes^33^,^34^. This phenomenon, more commonly known as the coral-probiotic hypothesis, states that stressful environmental conditions select for beneficial coral-microbe relationships^11^,^12^. Resident coral bacteria may prevent infection by producing antibiotics and/or by occupying space on the coral surface, thus preventing potential pathogens from colonizing^35^. Our study suggests that healthy CFCF may contain antimicrobial molecules that significantly inhibit the growth of the *Cytophaga-Flavobacterium* group (Fig. 2a). Diseased corals lose this ability, demonstrated by the proliferation of *Cytophaga-Flavobacterium* from healthy bacterial homogenates on plates infused with diseased CFCF (Fig. 2a). It is not yet clear whether those antimicrobials are derived from the coral host and/or its resident microbes^5^.

Unexpectedly, CFCF did not have a significant impact on *Vibrio* spp. growth. This may be attributed to a low abundance of culturable vibrios living within healthy *A. cervicornis* tissue (Fig. 2b). A high abundance of *Vibrio* spp. was observed within diseased coral homogenates, indicating that they are associated with the WBD state (Fig. 2b). Increases in *Vibrio* species living within diseased or bleached coral tissue is a relatively common observation^4^,^9^.

The AHL Addition Experiment may provide greater insight into *Vibrio* activity on *A. cervicornis*. Selective culturing of bacterial homogenates showed an apparent difference in chemical composition between healthy and diseased coral CFCF. Interestingly, the addition of QS chemical signaling molecules to healthy coral homogenates has the ability to convert a healthy-associated microbiome into a pathogenic vector. Specifically, incubation with exogenous N-Hexanoyl-DL-homoserine lactone can shift a healthy-associated coral homogenate into a disease-inducing agent. This is demonstrated by the significant difference in time to tissue loss between the corals in the Healthy Control versus the Healthy + AHL treatments (Fig. 3). The Healthy + AHL treatment showed a greater similarity to both the Disease Control and the Disease + AHL treatments as far as inducing WBD-like tissue loss in healthy *A. cervicornis* fragments (Fig. 3). This result suggests that the addition of a common AHL can transform a population of commensal bacteria into pathogens.

The acyl-homoserine lactone QS system is well characterized in many *Vibrio* species, including *Vibrio cholerae*, *Vibrio fischeri*, and *Vibrio harveyi*. *V. harveyi*, in particular, is closely related to a number of suspected and established marine pathogens from the *Vibrio* genus^18^,^27^,^36^. In addition, *V. harveyi* (synonym *V. charchariae*) has recently been implicated as a potential primary pathogen for WBD^37^,^38^. Three parallel QS systems regulate gene expression in *V. harveyi*: an AI-1 mechanism controlled by an AHL, the AI-2 mechanism controlled by the universal gram-negative autoinducer, and a Cqs system similar to the AI-1 mechanism which functions at lower cell densities^16^. Together these three systems control many functions including bioluminescence, type III secretion, and polysaccharide, siderophore, and metalloprotease production^16^.

In many marine vibrios, virulence is positively controlled by QS in similar pathways to those found in *V. harveyi*^15^,^16^,^31^. In other cases, QS negatively controls virulence as part of a late-stage infection strategy^20^,^39^. Many *Vibrio* species, including known marine pathogens *Vibrio parahaemolyticus* and *Vibrio vulnificus*, employ a combination of positive and negative QS regulation systems to control virulence-associated genes^16^,^39^,^40^. It is likely that QS in marine vibrios is also involved in repression of host immunity. *Pseudomonas aeruginosa*, a related gammaproteobacteria, is a well-studied opportunistic pathogen that has demonstrated an ability to disrupt host cells through extensive QS-controlled protease production^41^,^42^,^43^,^44^. This has profound implications for organisms—such as corals—that rely on protective mucosal barriers as part of their innate immunity. Although the connection between protease activity and coral disease has not been established, it is interesting to note that protease production occurs in *V. harveyi* and has been tied to successful *V. harveyi* infections of marine shrimp^45^,^46^.

Although lesser known than the gammaproteobacteria systems, species belonging to the Bacteroidetes phylum—which includes the *Cytophaga-Flavobacterium* group—also employ QS and are associated with marine diseases^30^,^47^. In many aquatic ecosystems, *Cytophaga-Flavobacterium* are chemoorganotrophs responsible for significant decomposition of dissolved organic matter (DOM)^48^. In addition, a biofilm-forming member of the *Cytophaga-Flavobacterium* group, *Tenacibaculum maritimum*, has recently been shown to produce both short- and long-chain AHLs similar to the molecules produced by vibrios^47^. The *Cytophaga-Flavobacterium* group also contains a number of known marine pathogens, including the aforementioned *T. maritimum*, which is responsible for the fish disease tenacibaculosis^47^,^49^,^50^,^51^. The preferred DOM energy source and newfound QS potential of *Cytophaga-Flavobacterium* gives credence to a number of possible outcomes regarding this group’s role in coral disease. *Cytophaga-Flavobacterium* growth within diseased CFCF could indicate that this group contributes to secondary infection by simply proliferating on decaying coral tissue (in line with the compromised-host hypothesis). However, the *Cytophaga-Flavobacterium* group also has the potential to emerge as a primary pathogen responding to AHLs of its own. Further studies are needed in order to determine the QS capabilities of the *Cytophaga-Flavobacterium* group on corals.

Overall, our results confirm that diseased CFCF has the ability to stimulate the growth of potential primary or secondary coral pathogens including *Cytophaga-Flavobacterium*. We also demonstrate that the addition of the autoinducer N-Hexanoyl-DL-homoserine lactone can convert a healthy coral homogenate into a disease-causing agent. In this study, exogenous AHL was able to convert a healthy microbial population into a disease vector that produced WBD-like symptoms in healthy *A. cervicornis* (Fig. 3). This result strongly suggests that autoinduction, via the addition of AHL, can manipulate QS pathways and their downstream genetic targets in coral-associated bacteria. Although we cannot be sure of the molecular mechanisms involved, it is clear that the addition of N-Hexanoyl-DL-homoserine lactone somehow influences the growth of coral-associated bacteria. Molecular characterization of CFCF and bacterial metatranscriptomic studies will reveal details regarding presence/absence of AHLs and coral-associated microbial gene expression. Furthermore, as bacterial communities vary greatly within and between ecosystems, these results should be corroborated by similar experiments throughout the Caribbean.

## Methods

### Coral Collection for Selective Culturing of Coral-Associated Bacteria Experiment

All corals were sampled in February 2014 from Crawl Cay (9° 14’ 00” N, 82° 08’ 00” W) in Bocas del Toro, Panama. Six healthy and six diseased (active WBD) *A. cervicornis* fragments (5 cm in length) were collected from 12 coral colonies (CFCF Group). In addition, three paired samples of healthy and diseased *A. cervicornis* fragments (also 5 cm in length) were collected from coral colonies displaying both the healthy and the WBD phenotype (Bacterial Homogenate Group). Coral fragments were transported to the Smithsonian Tropical Research Institute in separate containers and corals from the Bacterial Homogenate Group were acclimated in flow-through aquaria (separating diseased and healthy corals).

### Preparation of Healthy and Diseased Cell-Free Culture Fluid (CFCF)

Within two hours of collection, each healthy *A. cervicornis* fragment from the CFCF Group was separately homogenized in a sterile tube containing 10 mL of 0.2 μm-filtered seawater by vortexing with sterile 3 mm glass beads. The coral skeleton was then removed with sterile tweezers and the resulting coral tissue homogenates were pooled and filtered at 0.2 μm to obtain the healthy CFCF. Homogenates were pooled in order to ensure sufficient production of CFCF for 250 mL media. Differences among coral colonies were accounted for during the plating step when individual bacterial homogenates were plated onto media containing pooled CFCF. These steps were repeated for the diseased *A. cervicornis* fragments from the CFCF Group, resulting in diseased CFCF.

### Preparation of Media Containing CFCF

Selective media were created in order to cultivate certain bacterial genera. TCBS Agar (Thiosulfate Citrate Bile Salts Sucrose, from VWR International) and Cytophaga Media (from VWR International) were chosen to select for *Vibrio* spp. and *Cytophaga-Flavobacterium* spp. given their prevalence on diseased corals and strong association with WBD (based on Gignoux-Wolfsohn unpublished data)^52^. 500 mL of each type of media was prepared according to manufacturer instructions and split into two equal volumes of 250 mL. After media had cooled to 50 **°**C, 25 mL of each CFCF (healthy and diseased) was added to 250 mL of each media type. This resulted in the following four combinations of media and CFCF: (1) TCBS medium & healthy CFCF (2) TCBS medium & diseased CFCF (3) Cytophaga medium & healthy CFCF and (4) Cytophaga medium & diseased CFCF. All media/CFCF combinations were mixed before plates were poured.

### Plating of Healthy and Diseased *A. cervicornis* Bacterial Homogenates onto Media/CFCF

The paired healthy and disease *A. cervicornis* fragments from the Bacterial Homogenate Group were homogenized using the aforementioned vortexing technique. Start and end weights (before coral was added and after coral skeleton was removed) were taken of the tubes to normalize for grams of tissue plated in subsequent calculations. Each coral tissue homogenate was filtered at 5 μm to remove coral cells and symbiotic dinoflagellates. Based on initial dilution series, we decided to dilute the bacterial homogenates ten-fold in 0.2 μm-filtered seawater. 100 μL of each 1:10 dilution was added to each of the four media/CFCF combination plates and was spread using sterile 3 mm glass beads. Plates were incubated for 24 hours at 27 **°**C, after which photos were taken of each plate to record colony densities.

### Coral Collection for AHL Addition Experiment

Ten healthy and ten diseased (active WBD) *A. cervicornis* fragments (5 cm in length) were collected from 20 coral colonies to prepare bacterial homogenates (Homogenate Group). In addition, 12 fragments each (6 cm in length) were collected from three healthy *A. cervicornis* colonies to serve as the test coral fragments. Organisms were transported to the Smithsonian Tropical Research Institute in separate containers. For the test corals, three fragments from each of the three colonies (genotypes) were acclimated in four separate flow-through aquaria for several hours prior to the start of the experiment.

### Preparation of Healthy and Diseased Coral Homogenate and Addition of AHL

Within two hours of collection, each healthy *A. cervicornis* fragment from the Homogenate Group was separately homogenized in a sterile tube containing 10 mL of 0.2 μm-filtered seawater by vortexing as described above. The coral skeleton was then removed with sterile tweezers and the resulting ten healthy coral tissue homogenates were pooled. These steps were repeated for the ten diseased *A. cervicornis* fragments from the homogenate group. Both of the homogenate pools were split into equal halves of 50 mL and labeled as follows: “Healthy Control,” “Healthy + AHL,” “Disease Control,” and “Disease + AHL.” A 25 mM stock solution of the Proteobacteria autoinducer N-Hexanoyl-DL-homoserine lactone (Sigma Aldrich) was created using deionized water. This concentration was chosen to ensure a final 1.3 μM concentration of AHL in the aquaria. In other studies, AHLs were found to interact with cell membranes in the micromolar concentration range^53^,^54^,^55^. This particular AHL was chosen based on the length of its acyl chain as six carbon AHLs are produced by multiple gammaproteobacteria, including a number of known marine *Vibrio* pathogens^14^,^22^. The AHL solution was mildly heated and mixed until all AHL was fully dissolved. One mL of the AHL stock solution was then added to the “Healthy + AHL” and the “Disease + AHL” homogenates. One mL deionized water was added to the “Healthy Control” and the “Disease Control” homogenates. The four homogenates were incubated for five hours at room temperature with intermittent swirling.

### Lesioning and Dosing of Test Corals

Within the last hour of the AHL incubation period, all test coral fragments were lesioned by removing ~7.5 mm^2^ of tissue with an airbrush and 0.2 μm-filtered seawater to ensure waterborne disease transmission^3^. Immediately prior to dosing, the flow-through aquaria were converted to closed 20 L systems containing a water circulation pump. Each aquarium was then dosed with one of the four homogenates. After dosage, the experiment was checked every 12 hours and the health status of each coral fragment was recorded. We equated total tissue loss with coral mortality.

### Statistical Analyses

All statistical analyses were performed using R statistical software. For the Selective Culturing experiment, bacterial growth was measured by determining the percent bacterial coverage of each plate per mg of coral tissue using the point intercept method of counting in ImageJ^56^. Point intercept was used instead of traditional CFU counts in order to account for the fact that treatments containing diseased CFCF or diseased bacterial homogenates displayed a swarming phenotype. We believe this methodology does not alter the results as we found very significant percent coverage differences between diseased CFCF plates compared to healthy CFCF plates. Percent bacterial coverage per mg coral tissue plated was analysed with a two-way analysis of variance (ANOVA) that considered the CFCF in the media (healthy or diseased) and the plated bacterial homogenate (healthy or diseased) as fixed effects. The *Cytophaga-Flavobacterium* (Supplementary Table S1) and *Vibrio* (Supplementary Table S2) datasets were analysed separately. All ANOVA conditions were satisfied for the *Cytophaga-Flavobacterium* dataset. Levene’s test was used to test for homogeneity of variance (p = 0.5600) and the Shaprio-Wilk test was used to test for normality (p = 0.1991). In order to satisfy the ANOVA condition of homoscedasticity for the *Vibrio* dataset, percent bacterial coverage values were converted to a proportion. A small delta of 0.001 was added to each proportion to account for the zeros within the datasets. Values were then logit transformed^57^. Homogeneity of variance was satisfied (Levene’s test, p = 0.4320). The data were not normally distributed. All means were analysed using Tukey HSD post-hoc tests with an α value of 0.05.

For the AHL Addition experiment, time to total tissue loss in healthy test *A. cervicornis* fragments was analysed with a two-way ANOVA that considered the bacterial homogenate (healthy or diseased) and the addition or lack of AHL as fixed effects (Supplementary Table S3). Homogeneity of variance was satisfied (Levene’s test, p = 0.2327). The data were not normally distributed. All means were analysed using Tukey HSD post-hoc tests with an α value of 0.05.

## Additional Information

**How to cite this article**: Certner, R. H. and Vollmer, S. V. Evidence for Autoinduction and Quorum Sensing in White Band Disease-Causing Microbes on *Acropora cervicornis*. *Sci. Rep.* **5**, 11134; doi: 10.1038/srep11134 (2015).

## Supplementary Material

Supplementary Information
