# Supplementary material for: Evidence for Autoinduction and Quorum Sensing in White Band Disease-Causing Microbes on Acropora cervicornis
Source: Sci Rep. 2015 Jun 5;5:11134. doi: 10.1038/srep11134 (PMC4457150; doi:10.1038/srep11134)
Supplement: Supplementary Information [file srep11134-s1.pdf]

Evidence for Autoinduction and Quorum Sensing in White Band Disease-Causing Microbes on *Acropora cervicornis*

Rebecca H. Certner & Steven V. Vollmer

Supplementary Table S1

ANOVA table for *Cytophaga-Flavobacterium* dataset. Percent bacterial coverage per mg coral tissue plated was analysed with a two-way ANOVA that considered the CFCF in the media (healthy or diseased) and the plated bacterial homogenate (healthy or diseased) as fixed effects.

Source	*Df*	*Sum Sq.*	*Mean Sq.*	*F-value*	*P-value*
CFCF (cell-free culture fluid)	1	59,731	59,731	32.951	0.000434
Bacterial Homogenate	1	339	339	0.817	0.677033
CFCF:Bacterial Homogenate	1	3,738	3,738	2.062	0.188952
Residuals	8	14,502	14,502	-	-

Supplementary Table S2

ANOVA table for *Vibrio* dataset. Percent bacterial coverage per mg coral tissue plated was analysed with a two-way ANOVA that considered the CFCF in the media (healthy or diseased) and the plated bacterial homogenate (healthy or diseased) as fixed effects.

Source	*Df*	*Sum Sq.*	*Mean Sq.*	*F-value*	*P-value*
CFCF (cell-free culture fluid)	1	0.52	0.52	0.029	0.8697
Bacterial Homogenate	1	141.69	141.69	7.835	0.0232
CFCF:Bacterial Homogenate	1	0.52	0.52	0.029	0.8697
Residuals	8	144.67	18.08	-	-

Supplementary Table S3

ANOVA table for AHL Addition Experiment. Time to total tissue loss in healthy test *A. cervicornis* fragments was analysed with a two-way ANOVA that considered the bacterial homogenate (healthy or diseased) and the addition or lack of AHL as fixed effects.

Source	*Df*	*Sum Sq.*	*Mean Sq.*	*F-value*	*P-value*
Homogenate	1	309.17	309.17	2508	<2e-16
AHL	1	162.56	162.56	1319	<2e-16
Homogenate:AHL	1	180.01	180.01	1460	<2e-16
Residuals	32	3.94	0.12	-	-
